# Supplementary material for: Impact of Nitric Oxide on Polymorphonuclear Neutrophils’ Function
Source: Biomedicines. 2024 Oct 16;12(10):2353. doi: 10.3390/biomedicines12102353 (PMC11505631; doi:10.3390/biomedicines12102353)
Supplement: Supplementary file 1 [file biomedicines-12-02353-s001.zip › biomedicines-3186807-supplementary.pdf]

Supplementary Materials

Table S1. Results of the FACS measurement.

DHR fluorescence	Without NO (n = 8)	With NO (n = 9)
fMLP/TMF α [MFI]	21.7	42.8
PMA [MFI]	359.0	319.8
Surface epitopes	Without NO (n = 11)	With NO (n = 11)
CD11b [MFI]	322.0	359.5
CD62L [MFI]	309.3	293.1
CD66b [MFI]	264.5	293.4
